# A Review of Soil Contaminated with Dioxins and Biodegradation Technologies: Current Status and Future Prospects

**DOI:** 10.3390/toxics10060278

**Published:** 2022-05-24

**Authors:** Nguyen Thi Hong Nhung, Xuan-Tung Tan Nguyen, Vo Dinh Long, Yuezou Wei, Toyohisa Fujita

**Affiliations:** 1 School of Resources, Environment and Materials, Guangxi University, Nanning 530004, China; hnhung_2912@yahoo.com; 2 Faculty of Environmental and Food Engineering, Nguyen Tat Thanh University, Ho Chi Minh 700000, Vietnam; ntxtung@ntt.edu.vn; 3 Institute of Environmental Science, Engineering and Management, Industrial University of Ho Chi Minh City, Ho Chi Minh 700000, Vietnam; vodinhlong@iuh.edu.vn; 4 School of Nuclear Science and Technology, University of South China, Hengyang 421001, China; yzwei@usc.edu.cn; 5 School of Chemistry and Chemical Engineering, Guangxi University, Nanning 530004, China

**Keywords:** dioxins, soil, contamination, bioremediation, toxic

## Abstract

This article provides a comprehensive assessment of dioxins contaminating the soil and evaluates the bioremediation technology currently being widely used, and also offers recommendations for future prospects. Soil pollution containing dioxins is extremely toxic and hazardous to human health and the environment. Dioxin concentrations in soils around the world are caused by a variety of sources and outcomes, but the main sources are from the consequences of war and human activities. Bioremediation technology (bioaugmentation, biostimulation, and phytoremediation) is considered an optimal and environmentally friendly technology, with the goal of applying native microbial communities and using plant species with a high biomass to treat contaminated dioxins in soil. The powerful bioremediation system is the growth of microorganisms that contribute to the increased mutualistic and competitive relationships between different strains of microorganisms. Although biological treatment technology can thoroughly treat contaminated dioxins in soil with high efficiency, the amount of gas generated and Cl radicals dispersed after the treatment process remains high. Further research on the subject is required to provide stricter control over the outputs noted in this study.

## 1. Introduction

Dioxins are environmentally stable solid substances with high melting and boiling points and very low vapor pressure [1]. Dioxins are almost insoluble in water, have high thermal stability that can only be completely decomposed at temperatures above 1200 °C, and are resistant to strong acids and alkalis and adhere to the surface of organic resources, especially in soil [2,3]. In addition, dioxins are also substances which are man-made through activities such as the production of 2,4,5-T herbicides, chlorine-containing plant protection agents, combustion processes (the burning municipal waste, medical waste, industrial waste, especially waste containing PVC and metallurgical processes), and pulp bleaching with chlorine substances [4,5,6]. Dioxins are dangerous threat agents, even at low concentrations (one part per billion), which are enough to wreak havoc on human health and the environment [3]. In humans, dioxin exposure in humans effects the endocrine glands and reproductive functions, causes diseases in the central and peripheral areas of the nervous system, and impedes fetal development, especially in the nervous system and joints [7,8]. Dioxins can be remain in the environment for a long time, seeping deeply into soil and sediments [2]. Therefore, dioxins remaining in the soil and sediments will seep into water sources and ecosystems, including those that produce fish, shrimp, vegetables, and crops, posing a risk of poisoning for future generations [9].

Several studies have been conducted on physical, chemical, and biological dioxin remediation technologies. In physical treatment, physical processes, such as radioactive material degradation using solvent extraction and liquefied petroleum gas extraction methods [10,11], steam distillation [12], thermal desorption [13], and in situ vitrification [14] are used to degrade persistent organic pollutants,. Chemical reactions, such as basic catalytic decomposition, above and below extreme water treatment [15], photolysis at the spot level [16,17], electronic solvation technology [18], and K-polyethylene glycol technology [19] are used in chemical treatments to degrade persistent organic pollutants in the soil. Recently, nanotechnology has also been applied, using nanoscale zero-valent iron [20] to decompose persistent organic pollutants. Bioremediation aims to remove persistent organic compounds from contaminated soil using the anaerobic and aerobic decomposition of microorganisms [21,22]. Indigenous microorganisms enriched from dioxin-contaminated sites are believed to be able to remove dioxins [23,24]. The dechlorination of dioxins by microbial metabolism under anaerobic and aerobic conditions are the two main mechanisms of dioxin degradation [25]. Microbial strains can use dioxins as a carbon and energy source [26] to effectively dechlorinate dioxins against highly chlorinated congeners [27]. Phytoremediation is more effective and widespread, but only with the use of plants that have a large biomass, and is capable of dioxin adsorption in many laboratory and field studies [28,29].

There are many studies on airborne and food dioxins as the main sources in different countries, but there are not many general reports on dioxin-contaminated soil, especially for the bioremediation of dioxins in soil. Furthermore, this paper focuses on analyzing, comparing, and discussing a global overview of the situation of dioxin-contaminated soil. This review provides a comprehensive summary and discussion of relevant studies on dioxin-contaminated soil bioremediation. An assessment will contribute to the optimization parameters of bioaugmentation, biostimulation, and phytoremediation in the remediation of dioxin-contaminated soils. Current knowledge, research challenges/gaps, and prospects for future research are presented in this study.

## 2. Overview of Dioxins

**Figure 1:**
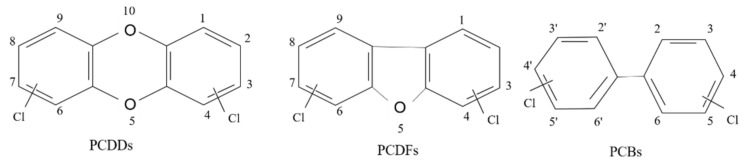
The basic structures of Polychlorinated dibenzo-p-dioxin (PCDD), dibenzofuran (PCDF), and biphenyls (PCBs).

Dioxins are persistent organic pollutants produced by both natural and human activities [30]. Dioxins are also the common name for a group of hundreds of chemical compounds that persist in the environment, as well as in the human body and other organisms [31]. Dioxins are very stable compounds with low polar, lipophilic, and hydrophobic qualities. They are classified into three groups: polychlorinated dibenzo-p-dioxins (PCDDs, referred to as dioxins), polychlorinated dibenzofurans (PCDFs, referred to as furans), and coplanar polychlorinated biphenyls (dioxin-like PCBs, referred to as dl-PCBs). The chemical structures of PCDDs, PCDFs, and PCBs are shown in Figure 1 [1]. Depending on the number of chlorine atoms and the spatial position, dioxins have 75 congeners PCDD (poly-chloro-dibenzo-dioxins) and 135 congeners PCDFs (poly-chloro-dibenzo-furans) with different toxicity. In 210 congeners, 17 congeners are known to be highly toxic because they can have chlorine atoms at positions 2, 3, 7, and 8, (at least) on the benzene ring [3]. Based on the international toxicity equivalence factor (I-TEF), 2,3,7,8-TCDD/TCDFs are known to be the most toxic compounds [32].

### 2.1. Sources, Fate, and Transportation of Dioxins in Soil

**Figure 2:**
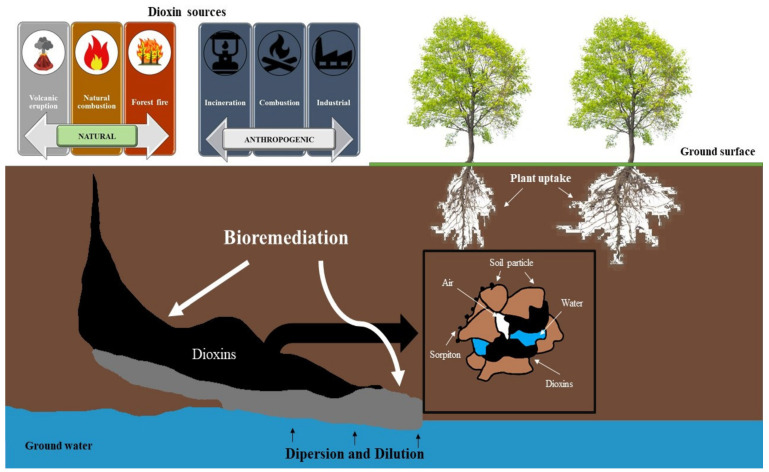
Sources, fate, and transportation of dioxins in soil.

Dioxin sources are typically found in both natural and anthropogenic environments (Figure 2). Dioxins are a typical examples of persistent organic compounds with extremely complex structures and many different toxicities. Dioxins are naturally emitted from volcanic eruptions [33], forest fires [34], and natural combustion [35]. However, previous studies have shown that the emission of dioxins in the environment is mainly caused by humans. The main anthropogenic origins of dioxins are classified into three sources: incineration, combustion, and industrial processes [30,36,37].

Dioxins in soil are typically solid and cling to soil particles [38]. The fate and transport of dioxins in soil are depicted in Figure 2. Dioxins undergo diffusion and dispersion, as well as biodegradation processes (bioaugmentation, biostimulation, and phytoremediation) [39]. In addition, the fate and transport of dioxins in the soil media is affected by soil characteristics (moisture content, soil texture, pH, and organic matter) and environmental conditions (ground surface, groundwater, plants, weather conditions, and biological activity) [40]. Since soil particles often adsorb dioxins based on their low mobility and biodegradability [38], understanding the fate and transportation of dioxins in contaminated soil is essential in developing bioremediation technologies.

### 2.2. Toxicity and Health Risk Assessment

The World Health Organization (WHO-2005) has recommended the standard exposure levels of dioxins as 1–4 picograms WHO-TEQ/kg of body weight per one day, or 0.07 ppt in blood; the general environmental limit in most countries is 1200 ppt TEQ in soil and 150 ppt in sediment [3]. The United States Environmental Protection Agency (US-EPA) considers reducing the dioxins limit to 72 ppt TEQ to increase the volume of contaminated soil to be treated [3]. Dioxins, as a dangerous threat agent to the environment and humans, are associated with severe damage to human health, shortening the lives of those exposed and potentially shortening the lives of their children and future generations [3]. When dioxin levels in humans exceed the allowable threshold of 0.0064 picograms/kg of the human body (US-EPA), dark patches of skin appear quickly as a result of cell death, mutated pigment cells, and impaired liver and kidney function [8,41]. If long-term exposure to levels exceeds the threshold, dioxins will affect the immune system, endocrine glands, and reproductive functions, leading to some cancers and diseases of the central and peripheral nervous systems, thyroid disorders, immune system damage, endometriosis, diabetes, etc. [8,42]. As a result, children of exposed parents were born with many tragic deformities, and some children lived in a vegetative state from birth; the association between dioxins exposure and five diseases, such as soft tissue cancer, benign lymphoma, chronic lymphocytic leukemia, hairy leukemia cancer, and chlorosis was also noted [2,43]. Some diseases associated with dioxin exposure, such as acute, chronic, and subacute peripheral neuropathy; chlorosis; type 2 diabetes; liver cancer; lipid metabolism disorders; reproductive abnormalities and birth defects, such as cleft lip and palate; congenital malformations of the legs, hydrocephalus, neural tube defects, adhesions (sticky fingers or toes), muscle malformations, and paralysis [43] have also been observed. The half-life of dioxins in the soil is from 60 to 80 years, and at the same time, it persists for a long time in the environment, seeps into the soil and sediments, and migrates into vegetation and aquatic life, leading to bioaccumulation in the soil and food chain [2,9,44].

## 3. Situation of Dioxin-Contaminated Soil and Standard Limits

Environmental pollution in general, and dioxins pollution in the soil in particular, are markedly on the rise. The main sources of dioxin emissions are industrial activities (such as combustion) which are an important part of human production. Dioxin emissions from G20 industrial activities account for more than 80% of the estimated annual emissions [41]. According to Miguel Dopico and Alberto Gómez et al., 2015, annual global dioxin emissions were 17,226 kg, equivalent to about 287 kg-TEQ. The main sources of dioxins in the soil environment are fuel combustion, metal production, pesticide production and use, waste incineration, accidental fires, landfill disposal, combustion, and herbicide runoff in agricultural uses [42].

Many countries around the world are currently hotspots for dioxin contamination, including Germany [43], Korea [44], the Mediterranean [45], South Africa [46], Poland [47], China [48], Vietnam [3,49], etc. In China, soil dioxin concentrations are primarily found in soils in the vicinity of production areas, such as urban, agricultural, and mountain soils [48]. Dioxin concentrations in soil in China have varied ranges at the provincial sampling points, listed in the Table 1 below, used to assess the concentration and health risks of people living in the area. According to research by Gene J. Zheng et al. in the mainland of China, Hong Kong, and Taiwan, the main source of PCDD/Fs pollution is from industrial waste activities, with total PCDD/Fs up to 967,500 ppt of dry weight in a soil sample located in the eastern part of Guangdong Province [50]. Soil samples in urban and rural areas in Liaoning province were also compared, and the results revealed that the concentration of dioxin-like PCBs in urban areas is higher than in rural areas [51]. In general, the dioxin concentrations in the east of China are lower than in the rest of China, and the urban and manufacturing areas are higher than the rural areas. While dioxins in the soil are primarily found in China due to industrial activities, high concentrations of dioxins have been found in Vietnam in areas affected by previous wars due to Agent Orange [49]. The majority of research documents on dioxin content in Japanese soil come from agricultural sources and incinerators; there have not been many studies published on this topic in recent years. A study of soil samples taken in China and Korea’s coastal areas revealed that the concentrations of PCDD/Fs at the sampling sites in Korea were higher than those in China, but both countries are lower than Japan [52].

Dioxin concentrations have not decreased in Europe in recent years, but instead, have increased significantly due to new dioxins emission sources, such as the illegal disposal of electric transformers in Italy [65] and Sweden [66]. Besides the main sources of pollution from manufacturing industries, motor vehicle emissions are one of the sources of pollution in Russia, because motor fuel combustion is dependent on the dopes used [67]. The results of dioxin concentrations in various regions of European countries are compared in Table 2; it is revealed that the concentration of dioxins in the soil in Spain, Slovakia, and Austria was lower than in other countries. Similarly, another study in Spain on dioxin levels affected by various sources, including municipal solid waste incineration, clinical waste incineration, and industrial areas, found results ranging from 0.45 to 14.41 ppt-TEQ (dry weight), with the effects of uncontrolled combustion processes being the most severe [68]. Research results on dioxin content in the soil in the UK by C.S. Creaser et al. showed that uncontrolled combustion leads to higher results in urban areas than in rural areas [69,70].

Similar to the results of searching for research documents on the situation of dioxin pollution in the soils of European countries, there are few data on the situation of soil research in America in recent years. In general, across the United States, dioxin concentrations in urban soil are generally higher than in rural soil, with maximum concentrations reaching 186 ng/kg according to TEQ in urban areas [74]. Another study conducted in Washington state discovered relatively low dioxin concentrations in soils, ranging from 0.14 to 4.1 ng/kg by TEQ, with agricultural land having the lowest dioxin value and urban land having the highest dioxin value, in accordance with other studies [75].

Research data on dioxins in countries in Africa are limited because the cost of dioxin analysis is expensive, and the analytical capacity is limited in this country [76]. According to research by C. Nieuwoudt et al., the dioxin concentrations in the soil of the sampled areas in Africa ranged from 0.34 to 20 ng/kg by TEQ, lower than those in Europe and the US [46,77]. The study also found that dioxin concentrations at industrial sites were higher than in agricultural soils and higher than in non-industrial sites, with combustion sources being the primary polluters [77].

Depending on soil pollution and effective land-use planning, several organizations around the world, such as the US EPA and WHO, have issued regulations on the concentration of pollutants in the soil (e.g., dioxins). However, some countries have developed industries in which the emission of pollutants into the soil is high (or have been affected by war, such as Vietnam), so each country will have its own regulations on PCDD/Fs concentrations in soil. The details are presented in Table 3.

## 4. Biodegradation Technologies of Dioxins

Biological treatment methods to reduce pollutants in different environments are considered effective and environmentally friendly [39]. Bioaugmentation is the addition of a degradation capacity into the soil to increase contaminant degradation, with effective potential for the bioremediation of organic-contaminated soils [85,86]. The addition of nutrients can encourage microbial activity by adjusting soil nutrients, which is known as biostimulation [87]. Composting is used to convert organic waste into simple organic substances. Bio-composting has traditionally been considered an eco-friendly remedy for organic soil contaminants, including petroleum, dioxins, and furans. Composting incubation is divided into mesophilic, thermophilic, cooling, and maturation stages, depending on microbial metabolism and heat production [88]. The mesophilic phase (<45 °C) occurs when the microbial community adapts to the initial conditions, and their numbers increase rapidly due to the readily degradable organic substrates [89]. Phytoremediation is frequently studied for its potential for immediate soil use with persistent organic compounds [28]. The main mechanisms involved in phytoremediation are based on the combined effects of plant uptake and the accumulation of toxic substances [90]. In this paper, bioremediation technology, including bioaugmentation, biostimulation, and phytoremediation, is analyzed and discussed below for the treatment of dioxin contamination in soil.

### 4.1. Bioaugmentation

Using microorganisms and fungus is currently an active application trend in dioxin-contaminated soil because of its low cost and environmental friendliness. The dechlorination of dioxins by microbial metabolism under anaerobic and aerobic conditions are the two main mechanisms of dioxin degradation in biological treatment [25]. Microbial strains can use dioxins as a carbon and energy source [26] to effectively dechlorinate dioxins from highly chlorinated isomers [27]. Table 4 lists microorganisms strains capable of degrading dioxins. Certain microorganisms such as *Pseudomonas, Mendocino*, and *Dehalococcoides* have been shown to effectively dechlorinate dioxins under anaerobic conditions [25,91,92]. Furthermore, aerobic microorganisms were discovered to degrade dioxins more efficiently and quickly than typical dioxin-contaminated soil anaerobes. Catechol 2,3-dioxygenase (C23O) is an important enzyme that catalyzes the reaction using molecular oxygen to destroy benzene rings [22], and *Bacillus* (*Firmicutes*) is the most dominant strain in aerobic degradation [93]. In addition to the strains of microorganisms that have been found to degrade dioxins with high efficiency, fungi also play a similar role, with their high mass and rapid environmental metabolism. Fungi are a diverse group of organisms, present in most environments and playing an integral role in ecosystems. In addition, fungi can regulate the flow of nutrients and energy through their network [89]. Furthermore, fungi are also unique organisms that can be used in the remediation of persistent organic wastes (POPs) in different environments, such as soil, water, and air [94]. Soils heavily contaminated with dioxins can also use fungi to decompose (with high efficiency) some typical strains such as *Cordyceps sinensis* strain *A* [15], *Phlebia lindtneri* [95,96], *Phanerochaete sordida YK-264* [97], etc. Table 5 presents some fungal strains capable of decomposing dioxins in soil, with high efficiency.

### 4.2. Biostimulation

Microbial adsorption highly contributes to the mineralization and co-transformation of organic contaminants during composting [115]. Therefore, microbial activity is the most important factor for the biodegradation of organic contaminants. In addition, the effects of operational parameters, such as humidity, growing conditions, and C/N ratio on the bio-incubation process are very important. Humidity is known as the main factor affecting the biodegradation of organic contaminants because it affects the microbial activity and the physicochemical properties of the contaminants [22]. Oxygen molecules participate in the catabolism and mineralization of hydrocarbon compounds by microbial and fungal activities. In addition, the C/N ratio plays an important role in the biodegradation of contaminants because it controls the composition of the microbial community. Previous studies have shown that the optimal C/N ratio for the biodegradation of organic contaminants by bio-incubation is between 10 and 40 [116]. Bio-composting has been successfully used in the biodegradation of dioxin-contaminated soils on a laboratory scale [25]. Chen et al., 2016, reported that the biodegradation efficiency of PCDD/PCDFs was approximately 95.8–99.7%, from an initial toxic concentration of 1580–3660 μg I-TEQ/kg dw after 42 days of incubation. Table 6 summarizes the organic compositions used to degrade dioxins in the soil by biological composting.

### 4.3. Phytoremediation

The application of phytoremediation is widely used by plants that grow in the wild, with features such as round and fat stems, many gaseous roots, or large roots, which can crawl on the ground or crawl on the trunk of another tree. These trees can grow, completely covering the ground, and remain green all year, are less deciduous, and yield a large biomass, which can withstand harsh environmental conditions, making it an ideal habitat for microorganisms and fungi in the rhizosphere, etc. They can form a favorable combination that optimizes the absorption and decomposition of toxic chemicals in the soil [119]. In addition, biological products, such as DECOM1 (a mixture of nutrient salts and organic humus), can be applied to the soil to increase the decay time of toxic substances to reduce the concentration of difficult pollutants, degrading, or in other words, helping plants absorb organic toxins more quickly in the soil [120,121]. Especially for the phytoremediation of persistent organic compounds in the soil, it takes a long time considering practical experimental conditions, such as weather, climate, other anthropogenic factors, etc. [122]. Table 7 below reviews some plants with the highest removal performance used for the treatment of dioxins in the soil.

## 5. Prospects for Future Research

Bio-composting can degrade dioxins in contaminated soil. However, to achieve high efficiency of the decomposition process, many different strains of microorganisms are required. Therefore, the biodegradation mechanisms of microbial strains are still poorly understood, and further research efforts are needed. To better understand the microbial diversity and structural changes associated with bio-composting, next-generation sequencing is proposed to identify the respective microbial strains. Lastly, the biodegradation process is designed to enhance the biodegradation process and shorten the processing time.

The growth and activity of microorganisms during the composting process determine the efficiency of the biodegradation of dioxins, which can be optimized through operational parameters, such as aeration rate, humidity, incubation time, pH, and C/N ratio. Optimal values of operational parameters vary with laboratory scale, pilot scale, composting material, and soil properties relative to the properties of dioxins. Currently, the knowledge from the literature is not sufficient to achieve commonly used optimal values. In addition, one of the major challenges of composting is that microbial activity is very time-consuming, which demonstrates why field-scale studies are very rare.

Additional studies are required to accurately and completely evaluate dioxin contamination sites of various origins and locations in order to provide effective treatment options. Furthermore, the bioremediation approaches for various contamination sites should be investigated. Other methods currently remain limited, such as hybrid bioremediation strategies in developing some transgenic plants to express dioxin-degrading enzymes, or nano-phytoremediation by combining the nanoparticles and vegetal species, which should be emphasized to improve the biodegradation efficiency of dioxins. In addition, the combination of chemical and biological measures or the combination of physicochemical and biological technologies should be utilized to improve efficiency in the degradation of pollutants.

Overall, future studies should provide more insight into the microbial relationships in the biodegradation of dioxins, in addition to the biodegradation mechanisms outlined above. The performance parameters also need to be studied more deeply for study scaling purposes.

## 6. Conclusions

With the current rate of industrial development and urbanization, the land area is shrinking, land quality is deteriorating, and land area per capita is decreasing. Currently, there are many hotspots of dioxin pollution in the soils of some countries around the world; the main source of dioxin-contaminated soil is industrial production activities, followed by the consequences of war, producing high dioxin concentrations and widespread infection. Dioxin contamination in the soil not only has a negative impact on industrial production, agriculture, and service activities, but it also has an indirect impact on human and animal health through food, vegetables, etc. Composting is full of economic benefits, it can treat dioxins in contaminated soil with high efficiency, and it is environmentally friendly. Parameters such as temperature, humidity, pH, oxygen content, aeration rate, and C/N ratio need to be continuously monitored and controlled during the composting process. The microbial community is primarily responsible for the biodegradation of dioxins in the soil. Some microbials can use dioxins as a source of carbon and energy to break down these compounds. The correlation between the microbial communities and the breakdown of dioxins during the composting process needs to be further studied so that the metabolic and congeners mechanisms can be elucidated. Ultimately, current knowledge is insufficient to achieve an optimal set of values for the treatment of soils contaminated with dioxins on a laboratory scale, pilot scale, and field scale.

## Figures and Tables

**Table 1:** The average concentration of dioxins (homogeneous unit calculated in ppt TEQ in dry weight) in some different nations in Asia.

Country	Year	Type of Soil	Source Area	Concentration	References
China (Sichuan)	2013	Soil	High mountain area	2.48–4.30 ppt	[53]
China (mainland Hong Kong and Tai wan)	2008	Soil	Schistosomiasis disease area	244.8–33,660 ppt	[50]
		Soil	E-waste recycling	799,000–967,500 ppt	[50,54]
		Paddy soil	E-waste recycling	2552–2726 ppt	[50]
		Soil	Pentachlorophenol manufacturing factory	606,000 ppt	[50,55,56]
South China	2022	Surface Soil	Municipal solid waste incinerator	114–2440 ppt	[57]
North China	2020	Soil	Urban green space in a metropolis	11.5–91.4 ppt	[58]
Eastern China	2009	Surface Soil	Electronic solid-waste with incinerators	0.017–5.04 ppt	[59]
North China	2011	Topsoil	Coastal areas	6.78–12.3 ppt	[52]
Central Vietnam	2019	Surface soil	The storage of Agent Orange in A-So Airbase during the Vietnam War	2.7 to 746 ppt	[60]
Southern Vietnam	2007	Topsoil	Bien Hoa Airbase was a former storage depot for Agent Orange	4.6–184 ppt	[61]
Japan (Osaka)	2013	Surface soil	Incineration plant	>1000 ppt	[62]
		Paddy field soil	Former herbicide use	38–110 ppt	
Japan (Akita)	2007	Paddy soil	Agricultural area	18,000–540,000 ppt	[63]
		Non-agricultural soil samples	Parks	950–1400 ppt	
South Korea	2021	Soil	Industrial sites	77.73 ppt	[64]
West Korea	2011	Topsoil	Coastal areas	14.2–27 ppt	[52]

**Table 2:** The average concentration of dioxins (homogeneous unit calculated in ppt TEQ in dry weight) in some different nations in Europe.

Country	Year	Type of Soil	Source Area	Concentration	References
Sweden	2013	Soil	Contaminated sawmill site	0.62–690,000 ppt	[66]
Russia	2011	Soil	Urban site	8.2 ppt	[67]
Poland	2015	Soil	Urban site	475.48–3039.27 ppt	[47]
Germany	2007	Soil	Alluvial flood plain of the river	7680 ppt	[57]
Spain	2006	Topsoil	High industrial activity zones	0.33–9.99 ppt	[71]
Slovakia	2012	Topsoil	Industrial site	0.34 to 18.05 ppt	[72]
Austria	2004	Soil	Agricultural site	0.05–23 ppt	[73]

**Table 3:** Some standards limitations for PCDD/Fs (ppt TEQ) in different nations.

National	Standard Limitation	Comments	Regulation/Guideline Values	References
US EPA Region 5	11 ppt 38.6 ppt	PCDD in soil PCDFs in soil	US EPA Region 5 ecological screening levels	[78]
US EPA Region 9	39 ppt	Residential soil	US EPA Region 9 preliminary remediation goal for 2,3,7,8-TCDD	[79]
China (Taiwan)	1000 ppt	General soil	The standard limit—Taiwan EPA	[80]
Vietnam	100 ppt 300 ppt 1200 ppt	Forest soil Agricultural soil Commercial soil	National technical regulation on the permissible limit of dioxins in soil	[81]
Finland	500 ppt	Agricultural and residential soil	Finland Ministry of the Environment, Department for Environmental Protection	[82]
Sweden	10 ppt 250 ppt	Land with sensitive use, Land with less sensitive use and groundwater extraction	Sweden Generic Guidance Value	[82]
Netherlands	10 ppt 1000 ppt	Dairy farming Agricultural and residential soil	The Netherlands Guidelines	[82]
Germany	5–40 ppt 100 ppt 1000 ppt 10,000 ppt	Agriculture Landscape Residential soil Industrial soil	Germany regulatory limit and recommendation	[82]
New Zealand	100 ppt 1500 ppt 18,000 ppt 90,000 ppt 21,000 ppt	Agricultural soil Residential soil Industrial soil Industrial-paved soil Maintenance	New Zealand Interim Acceptance Criteria	[83]
Canada	4 ppt	Alert soil	Canadian Environmental Quality Guidelines	[84]

**Table 4:** Bacterial strains capable of biodegrading dioxins in a soil matrix.

Bacterial Strains	PCDD/Fs Congeners	Concentration	Removal Average (%)	Time	References
*Terrabacter sp.* strain *DBF63*	2-CDD	10 μg/mL	75	18 h	[98]
	2,3-CDD		80		
	2-CDF		82.5		
	2,8-DCDF		85		
*Pseudomonas sp.* strain *CA10*	2-CDF		60		
*Pseudomonas sp.* strain *CA10*	2-CDD	1 μg/mL	97	5 d	[98]
	2,3-CDD		89		
*Sphingomonas sp.* strain *RW1*	DD	10 ppm	90	24 h	[99]
	2-CDD		90		
*Sphingomonas sp.* strain *KA1*	2-CDD	1 μg/g	96	7 d	[100,101]
	2,3-DCDD		70		
*Rhodococcus opacus SAO 101*	1-CDD	1 ppm	92	7 d	[102]
	Dioxin (DD)		97		
*Pseudomonas aeruginosa*	3,6-DCDF	10 mg/L	60	5 d	[103]
	1,2,3,4-TCDD		84		
	DBF		90		
*Pseudomonas veronii PH-03*	1-MCDD	1 μM	88.3	60 h	[104]
	2-MCDD		78.6		
	DD		90.7		
	DF		79.7		
*Sphingomonas sp. wittichi RW1*	DD	1 mM	81	72 h	[105]
	PCDD	29 ppt	75.5	15 d	
*Pseudomonas resinovorans* strain *CA10*	2,3-DCDD	1 μg/kg	90.95	7–14 d	[106]
*Pseudomonas resinovorans* strain *CA10*	2,3-DCDD	1000 μg/L	100	14 d	[106]
*Pseudomonas sp. CA10*	2-CDD	10,000 μg/L	98.5	7 d	[99]
*Pseudallescheria boydii*	2,3,7,8-TCDD	125 ng/g	92	15 d	[107]
*Stropharia rugosoannulata*	1,2,3,4,6,7,8-HpCDF	200 μg/L	64	3 m	[91]
*Bacillus-Firmicutes*	2,3,7,8-TCDD	136.33 ng/g	75	42 d	[108]
*Bosea BHBi7*	2,3,7,8-TCDD	170 ng/g	59.1	21 d	[109]
*Hydrocarboniphaga BHBi4*					
*Pseudomonas mendocina* NSYSU	OCDD	20.1 mg/kg	74	60 d	[92]

**Table 5:** Degradation of dioxins by fungi strains in soil matrix.

Fungi sp. Name	Pollutants Compounds	Nutrients/Conditions	Removal (%)	Time	References
*Cordyceps sinensis* strain *A*	DD	Glucose or 1,4-dioxane	50	4 d	[15]
	2,3,7-CDD		50		
	octaCDD		50		
*Phanerochaete sordida YK-624*	2,3,7,8-TetraCDD	Glucose	70	7 d	[97]
	1,2,3,7,7-PentaCDD		70		
	1,2,3,4,7,8-HexaCDD		75		
	1,2,3,4,6,7,8-HeptaCDD		70		
	1,2,3,4,6,7,8,9-OctaCDD		70		
	2,3,7,8-TetraCDF		45		
	1,2,3,7,8-PentaCDF		45		
	1,2,3,4,7,8-HexaCDF		75		
	1,2,3,4,6,7,8-HeptaCDF		70		
	1,2,3,4,6,7,8,9-OctaCDF		70		
*Acremonium sp.* strain *622*	T4CDD	Activated sludge and effluent	73	24 h	[110]
	P5CDD		85		
	H6CDD		79		
	H7CDD		76		
	O8CDD		88		
	T4CDF		81		
	P5CDF		88		
	H6CDF		84		
	H7CDF		84		
	O8CDF		71		
*Phanerochaete chrysosporium* strain *PcCYP11a3*	1-MCDD	Glucose	100	2 h	[111]
	2-MCDD		38.2		
	2,3-DCDD		6.1		
*Pleurotus pulmonarius* strain *BCRC36906*	HexaCDD/Fs	Solid state fermentation (SSF)	80	72 d	[112]
	HeptaCDD/Fs		97		
	OctaCDD/Fs		90		
*Phlebia radiata* strain *267*	1,2,3,4,7,8-H6CDD	Laccase, Tween-80 50 mL	28	30 d	[113,114]
	1,2,3,7,8-P5CDF		29		
	2,3,7,8-T4CDF		60		
*Phlebia radiata* strain *PL1*	1,2,3,7,8-P5CDD	Laccase, 50 mL Tween-80	76.3	30 d	[113,114]
	1,2,3,4,7,8-H6CDD		75.6		
	1,2,3,6,7,8-H6CDD		79.4		
	1,2,3,7,8,9-H6CDD		79.3		
	1,2,3,4,6,7,8-H7CDD		79		
	octaCDD		80		
	1,2,3,4,7,8-H6CDF		100		
	1,2,3,6,7,8-H6CDF		100		
	2,3,4,6,7,8-H6CDF		82.3		
	1,2,3,4,6,7,8-H7CDF		70.2		
	1,2,3,4,7,8,9-H7CDF		100		
	octaCDF		67.4		
*P. brevispora* strain *BMC3014*	2,7-DiCDD	Glucose and ammonium tartrate	33.8	14 d	[95,114]
	2,3,7-TriCDD		20		
	1,2,8,9-TetraCDD		15		
	1,2,6,7-TetraCDD		18		
*P. brevispora* strain *BMC9152*	2,7-DiCDD		54		
	2,3,7-TriCDD		30		
	1,2,8,9-TetraCDD		16.5		
	1,2,6,7-TetraCDD		26		
*P. brevispora* strain *BMC9160*	2,7-DiCDD		40		
	2,3,7-TriCDD		27		
	1,2,8,9-TetraCDD		23		
	1,2,6,7-TetraCDD		16.5		

**Table 6:** Summary of biodegradation statistics for dioxins in contaminated soil.

Initial Concentration	Mechanical Components	Materials	Removal (%)	Time (days)	Conditions	References
16,004 ng-TEQ/kg	Sandy loam	Food waste, sawdust, and compost	75	42	Aerobic	[108]
840–5300 ng-TEQ/kg	Sandy	Wood chips and compost	85	360	Semi-aerobic	[25]
30,000–60,000 ng-TEQ/kg	Sandy loam	Lime granules, Nutrients, and bark	21	175	Anaerobic	[117]
88.8–912.7 μmol/kg	Sandy loam	Sewage sludge	61.2	42	Aerobic	[32]
		Leaves	36.8			
		Animal manure	32.5			
		Sewage sludge and compost	53	280		
		Sewage sludge and animal manure	79			
6048 ng-TEQ/kg	Sandy loam	Food waste, sawdust, and compost	70	49	Aerobic	[118]
300–660 ngTEQ/kg	Sandy loam	Straw manure, bark chips, and wood chips	75	175	Semi-aerobic	[91]

**Table 7:** Degradation of dioxins by phytoremediation.

Names	Pollutant Compounds	Concentration	Removal (%)	Time	References
Arabidopsis thaliana	TCDD	10 ppt	72	30 d	[123]
		50 ppt	58		
		100 ppt	55		
Black Beauty	Total PCDDs	43 ppt-TEQ	46	32 d	[124]
	Total PCDFs		50		
Gold Rush	Total PCDDs	45 ppt-TEQ	60	32 d	
	Total PCDFs		62		
Spinach	Total PCDDs	3.42 ppt	48.6	ND	[125]
	Total PCDFs	0.519 ppt	37.9		
Garland Chrysanthemum	Total PCDDs	0.543 ppt	36.1		
	Total PCDFs	0.622 ppt	48.8		
Mitsuba	Total PCDDs	0.765 ppt	38		
	Total PCDFs	0.161 ppt	43.8		
Chingentsuai	Total PCDDs	0.268 ppt	39.2		
	Total PCDFs	0.166 ppt	41.6		
Rice leaf and stem	Total dioxins	317 ppt	90	5 m	[126]
Rice paddy chaff	Total dioxins	44 ppt	98		
Atena Polka	PCDD/Fs	7 ppt-TEQ dw	66	5 w	[127]
Zucchini	PCDD/Fs	155 ppt-TEQ dw	37	5 w	[127,128]
Cucumber	PCDD/Fs	122 ppt-TEQ dw	24	5 w	
Zucchini	2,4,8-TrCDF	0.0089 TSCF	64	4 d	[129,130]
	2,3,7,8-TeCDD		70		
Pumpkin	2,4,8-TrCDF	0.0064 TSCF	77		
	2,3,7,8-TeCDD		79		

## Data Availability

Not applicable.
